# Knowledge, attitudes, and practices regarding COVID-19 and mental health status among college students in China: a cross-sectional study

**DOI:** 10.3389/fpubh.2023.1157862

**Published:** 2023-06-28

**Authors:** Yi-Hua Li, Tao Wen, Yin-Shi Cui, Zhe-Hu Huang, Yan-Qun Liu

**Affiliations:** ^1^Department of Epidemiology, Department of Preventive Medicine, Department of Clinical Medicine, Medical College, Yanbian University, Yanji, China; ^2^Education and Scientific Research Office, Chaohanwen College, Yanbian University, Yanji, China; ^3^Affairs Management Department, Yanbian University, Hunchun, China; ^4^Department of Surgery, Affiliated Hospital of Yanbian University, Yanji, China; ^5^Orthopedic Treatment Center of Affiliated Hospital of Yanbian University, Yanji, China; ^6^Trauma Center of Affiliated Hospital of Yanbian University, Yanji, China

**Keywords:** knowledge, attitudes, practices, COVID-19, mental health

## Abstract

**Background:**

During the prevalence of coronavirus disease 2019 (COVID-19), little was known about the knowledge, attitudes, practices (KAP) about COVID-19 and psychological status of college students in minority areas. This study aimed to evaluate the KAP of college students in minority areas of China toward COVID-19 and to provide a scientific basis for health education and policy formulation.

**Methods:**

From October 28th to November 6th, 2021, a cross-sectional study was conducted with 5,272 college students to examine KAP and its effects on mental health.

**Results:**

Regarding COVID-19 knowledge, the overall awareness rate was 24.11% (1,271). Regarding health attitudes, most students had positive attitudes about COVID-19 prevention and control (94.95%), and females had higher positive attitudes than males (OR: 1.920; CI: 1.494–2.469). Regarding preventive behaviors, more than half of the students took preventive measures (53.48%), and freshmen had the highest health behavior scores. In terms of psychological status, there were fewer females with depression and stress than males.

**Conclusion:**

College students in minority areas have positive health attitudes; however, their knowledge of COVID-19 prevention and control is low. Moreover, their precautionary behaviors are insufficient, and they have many negative emotions.

## Introduction

An outbreak of coronavirus disease 2019 (COVID-19), caused by severe acute respiratory syndrome coronavirus-2 (SARS-CoV-2), occurred in Wuhan, China, in December 2019 and quickly spread worldwide. COVID-19 was listed as a public health emergency of international concern in January 2020 (1). The cumulative number of confirmed COVID-19 cases has reached 763.7 million, with the death toll exceeding 6.9 million, according to the WHO report on 19 April 2023 (2). As of April 19, 2023, a total of 99.2 million people have been diagnosed in China, and 120,912 have died from COVID-19. The strong infectivity, rapid spread, and wide infection range of COVID-19 seriously threatened people's safety and mental health; COVID-19 is one of the major public health emergencies experienced in China in the last hundred years, and its prevention and control present great challenges (3). Mass vaccination has become one of the most important public health policies for countries to prevent the spread of COVID-19. As of March 22, 2023, China has reported providing a total of 3.5 billion doses of COVID-19 vaccine. However, thus far, different variants of SARS-CoV-2, such as the Omicron variant, have been found to spread more widely and faster (4). Higher infection rates caused by mutation confirm that high vaccine coverage may not guarantee effective control of COVID-19 transmission. Therefore, the implementation of outbreak prevention and control measures cannot be ignored, which is closely related to public support (5).

The crisis caused by the COVID-19 pandemic is largely influenced by knowledge, attitudes and practices (KAP) (6). Knowledge, attitudes, and practices (KAP) are important cognitive components of health promotion in public health management, which greatly affects whether people follow preventive strategies and improve their behaviors as early as possible (7–9). Studies have found that knowledge of COVID-19 will affect people's attitudes and practices, while negative attitudes and practices will increase the risk of illness and death (10).

After the initial school closure and self-isolation period, universities reopened on September 7, 2020. However, due to the different severity of the COVID-19 pandemic in different provinces, the specific start time of the universities in different provinces is different (this study was conducted during the beginning of the third vaccination booster shot campaign in China). At present, the school has resumed classes and the order of teaching has been fully restored. The prevention and control of the COVID-19 pandemic on campus is facing new challenges. How to continue to do better in the prevention and control of campus pandemic and reduce the risk of students' physical and mental health affected by the pandemics has become an urgent public health problem (11). Despite daily prevention and control measures, such as maintaining social distance, frequent hand washing, and detecting suspected symptoms, universities were still at risk of outbreaks due to the large number of college students and their strong mobility. According to the statistics of education in 2021, the total number of students studying in higher education in China is 44.3 million. In addition, because it is unregulated, social media can provide misinformation, which can easily lead to ambiguity and misunderstandings regarding COVID-19 and even to panic and confusion (12). Studies have shown that college students' KAP level and mental health are affected by stressful events during the COVID-19 pandemic (13, 14), and the risk of anxiety, depression and other negative emotions among students increases, resulting in increased pressure (15–17). Therefore, good COVID-19-related knowledge, positive attitudes, healthy behaviors and good mental health are essential to effectively prevent and control pandemics and reduce the panic they cause (18–20).

The studied university is located in Yanbian ethnic Korean Autonomous Prefecture, a minority area in China, with 22,487 full-time college students. The time of questionnaire survey is in the normal prevention and control stage after the outbreak of COVID-19 in China. Yanbian University is a minority university and comprehensive university with strong ethnic regional characteristics. At the same time, it has the general characteristics of being a comprehensive university. Compared with universities in other regions, it has strong commonness and certain universality and representativeness. Investigating the knowledge, attitudes and practices of college students during the pandemic period is conducive to further understanding the weak points of pandemic prevention and control awareness in universities, so as to carry out accurate health education and prevention and control. From October 28th to November 6th, 2021, a questionnaire survey was conducted using the “Questionnaires” network platform to evaluate the KAP and mental health status of college students in China minority areas during the COVID-19 pandemic period and to analyze its influencing factors. This study provides a scientific basis for further health education and psychological intervention for college students' COVID-19 prevention and control and helps in the formulation of accurate prevention and control strategies to provide useful experience for curbing similar major public health emergencies in the future. The results are reported as follows.

## Materials and methods

### Study design

This cross-sectional survey was conducted from October 28th to November 6th, 2021 to evaluate the knowledge, attitudes, and practices about COVID-19 outbreak and psychological status of college students.

### Population and data collection

The research subjects were college students from a minority area in China. Considering the limitation of actual status in the sampling design and investigation implementation stage, non-probability sampling (convenient sampling) was adopted to conduct the current survey. Convenient sampling, also known as accidental sampling or natural sampling, is that researchers choose those who are easy to find or obtain information as the investigation objects according to the actual status. Therefore, expanding the sample size as much as possible can reduce the difference between the sample and the population. Therefore, in the implementation stage, this survey included a total of 5,272 large sample data to improve the representativeness of the sample. The effective response rate was 100%.

Referring to the Guide to the Prevention and Control of COVID-19 in Colleges and Universities (21) and authoritative official reports, we created an electronic questionnaire through the online survey platform “Questionnaires” and conducted a presurvey (a total of 300 people). After improving the questionnaire, we conducted a formal online survey through social media platforms (e.g., WeChat) from October 28th to November 6th, 2021. Instructions of the questionnaire on the front page clearly informed the participants of the research purposes and their rights regarding joining or dropping out of this study at any time during participation. All participants were informed and assured that their participation was voluntary, anonymous, and strictly confidential.

### Measures

The following tools were used in this study: (1) a general information questionnaire (sex, nationality, urban and rural areas, grade, major, etc.). (2) A self-made COVID-19 KAP questionnaire (COVID-19-related knowledge, attitudes and prevention practices), with 11 knowledge questions, receiving a total score of 11 points. And there are 10 health attitude questions. A 5-point Likert scale was used to measure and evaluate the support of COVID-19 prevention and control (1 = totally disagree to 5 = totally agree), with a total score of 50 points. Regarding health practice, five questions received scores of up to four points, and two questions received scores of up to three points, with a total possible score of 26 points. The study used a score of 80% as the cutoff. In terms of health knowledge, a score of ≥9 was considered high cognition, which was defined as awareness. In terms of health attitudes, a score of ≥40 was classified as positive health attitudes. In terms of health practice, a score of ≥21 was classified as good health behavior. (3) The Depression-Anxiety-Stress Scale (DASS-21) included 21 items divided into three dimensions: Depression, anxiety and stress. A stress scale score of >14 indicated psychological stress, an anxiety scale score of >7 indicated psychological anxiety, and a depression scale score of >9 indicated psychological depression. The scoring method of Bi et al. (22) was used. Those who had depression, anxiety and stress were classified as having negative emotions.

### Data analysis

SPSS 26.0 statistical software was used for statistical analysis of the data. Descriptive statistical analysis was performed on the number distribution of college students according to demographic characteristics. The count data are expressed by the composition ratio. Percentage comparisons were tested by binary logistic regression analysis to evaluate the related influencing factors. The variables of influencing factors in the results of logistic analysis are to explain the risk factors that affect the knowledge, attitudes, practices of COVID-19 prevention and control and psychological status among college students. For measurement data comparisons, *T*-tests were used for two groups and ANOVA was used for three or more groups, as described by[[Inline Image]]. A two-sided test was adopted, with a test level of α = 0.05, and the difference was considered statistically significant when *P* < 0.05.

### Ethical considerations

Informed consent was obtained from all subjects involved in the study. The study was conducted according to the guidelines of the Declaration of Helsinki, and approved by the Institutional Review Board of Yanbian University (Ethical Code: 2021576, October 26th, 2021).

## Results

### Basic information

A total of 5,272 college students were included in this study, and their general characteristics are shown in Table 1.

**Table 1:** Respondents' demographic information.

**Participants**		**No**.	**Cumulative percentage (%)**
Gender	Male	1,824	34.6
	Female	3,448	65.4
Ethnic group	Han	3,590	68.1
	Korean	1,151	21.8
	Other nationality	531	10.1
Town and country	Town	4,218	80.0
	Country	1,054	20.0
Grade	Freshman	2,677	50.8
	Sophomore	1,474	28.0
	Junior	641	12.2
	Senior	394	7.5
	Five-grade	86	1.6
Major	Medical major	1,253	23.8
	Non-medical major	4,019	76.2
Knowledge score	< 9	4,001	75.9
	≥9	1,271	24.1
Attitude score	< 40	266	5.0
	≥40	5,006	95.0
Practice score	< 21	2,454	46.5
	≥21	2,818	53.5
Psychological status	Bad	2,398	45.5
	Good	2,874	54.5

### College students' awareness of COVID-19

The average COVID-19 prevention and control knowledge score of college students was 7.07 ± 2.01. Regarding college students' knowledge about COVID-19, college students had good knowledge about the attributes of COVID-19 virus pathogens (71.9%) but insufficient knowledge about the pathogens (51.9%). The awareness rate of survival conditions (41.5%) and preservation conditions of COVID-19 nucleic acid test specimens (39.3%) was low. College students (95.0%) had a high degree of knowledge of COVID-19 transmission channels. The awareness rate of symptoms such as fever and dyspnea after COVID-19 infection was good (86.5%); however, the awareness rate of whether diarrhea can be the first symptom of COVID-19 was low (56.3%). College students had poor knowledge of the correct way to cover their nose and mouth when coughing or sneezing (19.3%). The students had good knowledge of the medical observation period of close contact home isolation for COVID-19 (88.8%). The awareness rate of wearing (84.9%) and taking off (71.8%) disposable medical masks was good (see Table 2).

**Table 2:** Knowledge of COVID-19 among college students.

**Item**	**Awareness rate (%)**
COVID-19 transmission route	5,007 (95.0)
Medical observation period of close contact home isolation in COVID-19	4,684 (88.8)
Symptoms after COVID-19 infection (fever, dyspnea, etc.)	4,560 (86.5)
Wearing mode of disposable medical mask	4,476 (84.9)
What kind of virus does COVID-19 belong to?	3,791 (71.9)
Removing method of disposable medical mask	3,786 (71.8)
Can diarrhea be the first symptom of COVID-19?	2,968 (56.3)
COVID-19 pathogen	2,734 (51.9)
COVID-19 is more likely to spread where pathogens survive for a long time	2,189 (41.5)
Preservation conditions of COVID-19 nucleic acid samples that can be detected within 24 h	2,072 (39.3)
How do you cover your nose and mouth when coughing or sneezing?	1,019 (19.3)

The overall awareness rate of COVID-19 prevention and control knowledge was low, accounting for 24.11% of the sample (1,271 people). Univariate analysis showed that there were differences in the awareness rate of COVID-19 prevention and control knowledge among college students of different genders, grades and majors (*P* < 0.05), and the results are shown in Table 3. Specifically, females had higher awareness than males. Five-grade students had the highest awareness than students in other grades. Medical majors had significantly higher awareness than non-medical majors.

**Table 3:** Single factor analysis of the awareness rate of COVID-19 prevention and control knowledge among college students.

**Participants**		***n* (%)**	**χ^2^**	***P*-value**
Gender	Male	410 (22.5)	4.052	0.044
	Female	861 (25.0)	–	–
Ethnic group	Han	887 (24.7)	2.363	0.307
	Korean	266 (23.1)	–	–
	Other nationality	118 (22.2)	–	–
Town and country	Town	1,041 (24.7)	3.766	0.052
	Country	230 (21.8)	–	–
Grade	Freshman	713 (26.6)	25.096	< 0.001
	Sophomore	325 (22.0)	–	–
	Junior	141 (22.0)	–	–
	Senior	68 (17.3)	–	–
	Five-grade	24 (27.9)	–	–
Major	Medical major	366 (29.2)	23.379	< 0.001
	Non-medical major	905 (22.5)	–	–

Logistic regression analysis was performed for the variables with statistical significance in the single factor analysis of the knowledge awareness rate, and the results were consistent with those of the single factor analysis. Multivariate regression analysis showed that females were the protective factors for the high awareness rate of prevention and control knowledge in COVID-19 (OR = 1.172, 95% CI = 1.024–1.341). Compared with freshmen, sophomores (OR = 0.787, 95% CI = 0.677–0.915) and juniors (OR = 0.793, 95% CI = 0.645–0.975) are the risk factors for high awareness (see Table 4).

**Table 4:** Binary logistic regression analysis of influencing factors of COVID-19 prevention and control knowledge among participants (*n* = 5,272).

**Participants**		**β**	**Wald χ^2^**	***P*-value**	**OR (95% CI)**
Gender	Male^*^	–	–	–	–
	Female	0.158	5.278	0.022	1.172 (1.024, 1.341)
Grade	Freshman^*^	–	–	–	–
	Sophomore	−0.239	9.663	0.002	0.787 (0.677, 0.915)
	Junior	−0.232	4.842	0.028	0.793 (0.645, 0.975)
	Senior	−0.515	13.367	< 0.001	0.597 (0.453, 0.787)
	Five-grade	0.030	0.014	0.904	1.030 (0.637, 1.665)
Major	Medical major^*^	–	–	–	–
	Non-medical major	−0.324	19.502	< 0.001	0.724 (0.627, 0.835)

^*^as the control group.

### College students' attitudes regarding the prevention and control of COVID-19

The average score of college students' attitudes toward COVID-19 prevention and control was 47.54 ± 5.70. More college students in minority areas have positive health attitudes (see Table 5). Univariate analysis showed that there were differences in the scores of COVID-19 prevention and control attitudes among college students of different sexes, nationalities, grades and majors (*P* < 0.05), and the results are shown in Table 6. Specifically, females had higher attitude scores than males. Students of Han nationality had higher attitude scores than students of other nationalities. Students in their first year had higher attitude scores than students in other years, and those in their second year had the worst attitude scores. Medical majors had lower attitude scores than non-medical majors.

**Table 5:** Attitudes of COVID-19 among college students.

**Items**	**Negative perception (%)**
In the last two months, if you have symptoms such as continuous fever, cough and runny nose, it is necessary to go to the hospital.	301 (5.7)
At this stage, do you think it is necessary to pay close attention to the news related to the COVID-19 epidemic?	255 (4.8)
At present, although the epidemic situation in China has been effectively controlled, it is still necessary to wear masks when going out to crowded places.	195 (3.7)
Prevention and control of the COVID-19 epidemic is not only a matter for the government, but also closely related to me personally.	229 (4.3)
Do you support quarantine measures for people who come into contact with suspected patients?	196 (3.7)
If the epidemic breaks out again, do you support stopping school and work in time?	320 (6.1)
Do you actively discourage friends and relatives from eating hunted meat now?	353 (6.7)
At present, the epidemic situation has been effectively controlled, and it is still necessary to inoculate COVID-19 vaccine.	177 (3.4)
COVID-19 vaccine is safe and effective.	349 (6.6)
Do you support the nucleic acid detection measures for the cross-regional flow of the public?	292 (5.5)

**Table 6:** Scores of college students' attitudes about COVID-19 prevention and control.

**Participants**		**Mean score ±SD**	***t*(*F*)**	***P*-value**
Gender	Male	46.92 ± 6.76	5.305	< 0.001
	Female	47.87 ± 5.02	–	–
Ethnic group	Han	47.70 ± 5.67	5.919	0.003
	Korean	47.02 ± 5.95	–	–
	Other nationality	47.66 ± 5.23	–	–
Town and country	Town	47.59 ± 5.73	1.033	0.302
	Country	47.38 ± 5.57	–	–
Grade	Freshman	48.02 ± 4.62	9.766	< 0.001
	Sophomore	46.88 ± 6.94	–	–
	Junior	47.22 ± 6.24	–	–
	Senior	47.35 ± 5.76	–	–
	Five-grade	47.33 ± 6.45	–	–
Major	Medical major	46.97 ± 6.58	3.707	< 0.001
	Non-medical major	47.72 ± 5.38	–	–

Most college students had positive health attitudes about COVID-19 prevention and control, accounting for 94.95% of the sample (5006). Logistic regression analysis was performed for the variables with statistical significance in single factor analysis of attitude scores, and the results were consistent with those of single factor analysis. Logistic regression analysis showed that females were the protective factors to keep positive perception on prevention and control of COVID-19 (OR = 1.920, 95% CI = 1.494–2.469). Compared with Han nationality, ethnic Korean nationality is a risk factor for maintaining a positive awareness of prevention and control in COVID-19 (OR = 0.633, 95% CI = 0.475–0.843). Compared with freshmen, other grades are risk factors for maintaining a positive attitude. Compared with medical majors, non-medical majors are the protective factors for maintaining a positive attitude (OR = 1.622, 95% CI = 1.268–2.177) (see Table 7).

**Table 7:** Binary logistic regression analysis of influencing factors of college students' attitudes toward COVID-19 prevention and control.

**Participants**		**β**	**Wald χ^2^**	***P*-value**	**OR (95% CI)**
Gender	Male^*^	–	–	–	–
	Female	0.653	25.909	< 0.001	1.920 (1.494, 2.469)
Ethnic group	Han^*^	–	–	–	–
	Korean	−0.457	9.783	0.002	0.633 (0.475, 0.843)
	Other nationality	−0.384	3.613	0.057	0.681 (0.458, 1.012)
Grade	Freshman^*^	–	–	–	–
	Sophomore	−0.989	43.162	< 0.001	0.372 (0.277, 0.500)
	Junior	−0.732	12.838	< 0.001	0.481 (0.322, 0.718)
	Senior	−0.968	17.505	< 0.001	0.380 (0.241, 0.598)
	Five-grade	−1.206	10.345	0.001	0.299 (0.144, 0.624)
Major	Medical major^*^				
	Non-medical major	0.508	13.577	< 0.001	1.622 (1.268, 2.177)

^*^as the control group.

### College students' COVID-19 prevention and control practices

The average COVID-19 prevention and control practice score of college students was 20.48 ± 4.46. Most college students have taken the prevention and control measures in COVID-19. However, there are still many college students who have not taken preventive practice in using public chopsticks, exercising and keeping social distance (see Table 8).

**Table 8:** Preventive practices of COVID-19 among college students.

**Items**	**Untaken preventive practice**
After returning to school, did you wear a mask in the place where many people studied (or worked) together?	1,561 (29.6)
In the last two months, did you wear a mask in crowded public places (such as supermarkets, shopping malls, buses, etc.)?	1,096 (20.8)
In the last two months, did you keep a distance when talking with others?	1,885 (35.8)
In the last two months, many people have eaten at the same table. Did you use public chopsticks?	2,060 (39.1)
Have you done any physical exercise in the last two months?	2,062 (39.1)
In the last two months, did you and your family buy or stock disinfection products or masks?	722 (13.7)
After the epidemic, if you have symptoms such as coughing or sneezing, will you consciously wear a mask?	527 (10.0)

The results of single factor analysis showed that there were significant differences in the scores of health practices for COVID-19 prevention and control among students with different urban and rural areas and grades (*P* < 0.05). More than half of the college students had good health behaviors, accounting for 53.48% of the sample (2818). Logistic regression analysis was further performed for the variables with statistical significance in the single factor analysis of practice scores. The results showed that, compared with freshmen, sophomores (OR = 0.601, 95% CI = 0.529–0.683), juniors (OR = 0.596, 95% CI = 0.501–0.709) and seniors (OR = 0.670, 95% CI = 0.542–0.828) were the risk factors for taken COVID-19 prevention and control behaviors. The results are shown in Tables 9, 10.

**Table 9:** Scores for COVID-19 prevention and control practices.

**Participants**		**Mean score ±SD**	***t*(*F*)**	***P*-value**
Gender	Male	20.33 ± 4.69	1.711	0.087
	Female	20.56 ± 4.33	–	–
Ethnic group	Han	20.51 ± 4.51	0.366	0.694
	Korean	20.38 ± 4.32	–	–
	Other nationality	20.47 ± 4.40	–	–
Town and country	Town	20.54 ± 4.56	2.032	0.042
	Country	20.23 ± 4.45	–	–
Grade	Freshman	21.07 ± 4.21	26.524	< 0.001
	Sophomore	19.77 ± 4.56	–	–
	Junior	19.71 ± 4.72	–	–
	Senior	20.26 ± 4.66	–	–
	Five-grade	21.06 ± 4.70	–	–
Major	Medical major	20.34 ± 4.39	1.302	0.193
	Non-medical major	20.52 ± 4.48	–	–

**Table 10:** Binary Logistic regression analysis of influencing factors for COVID-19 prevention and control practices.

**Participants**		**β**	**Wald χ^2^**	***P*-value**	**OR (95% CI)**
Town and country	Town^*^	–	–	–	–
	Country	−0.077	1.211	0.271	0.926 (0.808, 1.062)
Grade	Freshman^*^	–	–	–	–
	Sophomore	−0.510	60.728	< 0.001	0.601 (0.529, 0.683)
	Junior	−0.517	34.123	< 0.001	0.596 (0.501, 0.709)
	Senior	−0.401	13.724	< 0.001	0.670 (0.542, 0.828)
	Five-grade	0.204	0.800	0.372	1.226 (0.784, 1.917)

^*^as the control group.

### Mental health status of college students during the COVID-19 pandemic

During the pandemic period, there were 2,398 college students (45.50%) with negative emotions. The results of single factor analysis showed that there were significant differences in the proportion of depression among college students of different sexes and with different grades, knowledge, attitudes and practices (*P* < 0.05). There were significant differences in the proportion of anxiety symptoms among college students of different nationalities and with different grades, knowledge, attitudes and practices (*P* < 0.05). There were significant differences in the proportion of stress emotions among students of different sexes and nationalities and with different grades, attitudes and practices (*P* < 0.05). In the single factor analysis of negative emotions, the variables with statistical significance were further analyzed by logistic regression, and the results were consistent with those of the single factor analysis.

Logistic results showed that females were the protective factor for depression (OR = 0.799, 95% CI = 0.701–0.911). Compared with freshmen, sophomores (OR = 1.776, 95% CI = 1.534–2.056), juniors (OR = 1.506, 95% CI = 1.237–1.834) and seniors (OR = 1.763, 95% CI = 1.394) are risk factors for depression. Positive attitudes (OR = 0.497, 95% CI = 0.384–0.643) and healthy behaviors (OR = 0.570, 95% CI = 0.502, 0.648) are the protective factors for depression. In terms of anxiety, compared with freshmen, sophomores (OR = 1.790, 95% CI = 1.569–2.042), juniors (OR = 1.760, 95% CI = 1.474–2.100) and seniors (OR = 1.890, 95% CI = 1.521–2.348) are risk factors. Compared with Han nationality, ethnic Korean nationality (OR = 1.171, 95% CI = 1.020–1.344) and other ethnic minorities (OR = 1.280, 95% CI = 1.061–1.544) are risk factors for anxiety. Positive cognition (OR = 0.737, 95% CI = 0.570–0.952) and adopting healthy behavior (OR = 0.567, 95% CI = 0.507–0.635) are protective factors. As for stress, females are the protective factors (OR = 0.688, 95% CI = 0.549–0.863). Compared with freshmen, sophomores (OR = 1.914, 95% CI = 1.471–2.489), juniors (OR = 1.599, 95% CI = 1.120–2.281) and seniors (OR = 2.535, 95% CI = 1.741) are risk factors. Compared with Han nationality, ethnic Korean nationality is a risk factor for stress (OR = 1.424, 95% CI = 1.098–1.848). Maintaining a positive attitude (OR = 0.312, 95% CI = 0.226–0.433) and healthy behavior (OR = 0.466, 95% CI = 0.369–0.590) are protective factors for stress (see Tables 11, 12 for details).

**Table 11:** Univariate analysis of depression, anxiety, and stress of college students with different characteristics.

**Participants**		**Depression (%)**	**Anxiety (%)**	**Stress (%)**
Gender	Male	529 (29.0)	760 (41.7)	156 (8.6)
	Female	846 (24.5)	1,507 (43.7)	196 (5.7)
	χ^2^	12.343	2.025	15.750
	*P*-value	< 0.001	0.155	< 0.001
Ethnic group	Han	911 (25.4)	1,502 (41.8)	213 (5.9)
	Korean	324 (28.1)	512 (44.5)	97 (8.4)
	Other nationality	140 (26.4)	253 (47.6)	42 (7.9)
	χ^2^	3.502	7.685	10.143
	*P*-value	0.174	0.021	0.006
Town and country	Town	1,083 (25.7)	1,798 (42.6)	293 (6.9)
	Country	292 (27.7)	469 (44.5)	59 (5.6)
	χ^2^	1.800	1.204	2.462
	*P*-value	0.180	0.273	0.117
Grade	Freshman	548 (20.5)	953 (35.6)	118 (4.4)
	Sophomore	489 (33.2)	757 (51.4)	134 (9.1)
	Junior	190 (29.6)	325 (50.7)	48 (7.5)
	Senior	129 (32.7)	205 (52.0)	44 (11.2)
	Five-grade	19 (22.1)	27 (31.4)	8 (9.3)
	χ^2^	96.172	135.163	50.284
	*P*-value	< 0.001	< 0.001	< 0.001
Major	Medical major	330 (26.3)	546 (43.6)	78 (6.2)
	Non-medical major	1,045 (26.0)	1,721 (42.8)	274 (6.8)
	χ^2^	0.056	0.221	0.538
	*P*-value	0.813	0.638	0.463
Knowledge score	< 9	1,072 (26.8)	1,756 (43.9)	270 (6.7)
	≥9	303 (23.8)	511 (40.2)	82 (6.5)
	χ^2^	4.365	5.342	0.136
	*P*-value	0.037	0.021	0.712
Attitude score	< 40	123 (46.2)	149 (56.0)	59 (22.2)
	≥40	1,252 (25.0)	2,118 (42.3)	293 (5.9)
	χ^2^	59.053	19.358	108.063
	*P*-value	< 0.001	< 0.001	< 0.001
Practice score	< 21	805 (32.8)	1,262 (51.4)	237 (9.7)
	≥21	579 (20.2)	1,005 (35.7)	115 (4.1)
	χ^2^	107.616	132.970	65.471
	*P*-value	< 0.001	< 0.001	< 0.001

**Table 12:** Binary logistic regression analysis of influencing factors for college students' bad emotions.

**Participants**		**Depression**		**Anxiety**		**Stress**	
		***P*** **-value**	**OR (95% CI)**	***P*** **-value**	**OR (95% CI)**	***P*** **-value**	**OR (95% CI)**
Gender	Male^*^	–	–	–	–	–	–
	Female	0.001	0.799 (0.701, 0.911)	–	–	0.001	0.688 (0.549, 0.863)
Grade	Freshman^*^	–	–	–	–	–	
	Sophomore	< 0.001	1.776 (1.534, 2.056)	< 0.001	1.790 (1.569, 2.042)	< 0.001	1.914 (1.471, 2.489)
	Junior	< 0.001	1.506 (1.237, 1.834)	< 0.001	1.760 (1.474, 2.100)	0.010	1.599 (1.120, 2.281)
	Senior	< 0.001	1.763 (1.394, 2.230)	< 0.001	1.890 (1.521, 2.348)	< 0.001	2.535 (1.741, 3.691)
	Five-grade	0.814	1.065 (0.629, 1.803)	0.419	0.824 (0.516, 1.316)	0.064	2.069 (0.959, 4.461)
Ethnic group	Han^*^	–	–	–	–	–	–
	Korean	–	–	0.025	1.171 (1.020, 1.344)	0.008	1.424 (1.098, 1.848)
	Other nationality	–	–	0.010	1.280 (1.061, 1.544)	0.099	1.345 (0.946, 1.913)
Knowledge score	< 9^*^	–	–	–	–	–	–
	≥9	0.577	0.958 (0.824, 1.114)	0.444	0.950 (0.832, 1.084)	–	–
Attitude score	< 40^*^	–	–	–	–	–	–
	≥40	< 0.001	0.497 (0.384, 0.643)	0.020	0.737 (0.570, 0.952)	< 0.001	0.312 (0.226, 0.433)
Practice score	< 21^*^	–	–	–	–	–	–
	≥21	< 0.001	0.570 (0.502, 0.648)	< 0.001	0.567 (0.507, 0.635)	< 0.001	0.466 (0.369, 0.590)

^*^as the control group.

## Discussion

At present, the COVID-19 pandemic situation in China has been controlled, and it has entered the stage of normalized prevention and control (23). In addition to vaccines, strict preventive measures are the only other option to stop the rapid spread of diseases (24). Preventive measures play a vital role in reducing the infection rate of infectious diseases and controlling their spread, which suggests that it is necessary for the public to observe preventive measures, and the public's compliance with preventive measures is influenced by their KAP (25). Some studies have pointed out that people's KAP, as a part of public health emergency response capabilities, plays an important role in controlling the spread of COVID-19 (26). A large number of empirical studies have shown that health education can improve knowledge levels and change negative attitudes and behaviors, such as maintaining social distance, avoiding group gatherings and shaking hands, thus effectively curbing the spread of infectious diseases (27, 28). Empirical research on KAP can reveal basic information to determine the type of intervention to effectively control infectious diseases. Therefore, improving KAP is a potentially valuable strategy to gain better insight into misconceptions (29). Studies have found that in regard to health-related issues, college students can be a source of health awareness and health education because they are involved in spreading knowledge about the prevention and control of infectious diseases to the public for better tackling the ongoing pandemic (30, 31). In addition, a study found that social isolation and lockdowns due to the pandemic caused a series of mental health problems, such as stress, fear, anxiety, insomnia and emotional exhaustion (32). The evolution of the COVID-19 pandemic is uncertain and may have a long-term impact on mental health. College students with common mental health disorders are a vulnerable group. College students are in the delicate process of “transitioning” to adulthood and starting their careers, which makes them more prone to negative emotions (33). This study is helpful in bridging the gap in college students' knowledge, attitudes and behaviors regarding COVID-19 and improving their psychological states, which may reveal potential obstacles affecting changes in college students' social behavior.

### Knowledge, attitudes and practices of college students regarding COVID-19

The results of this study showed that the COVID-19 knowledge awareness rate was low (24.11%). Most college students have poor knowledge of COVID-19 prevention and control and a poor understanding of COVID-19 infection. The reason for this phenomenon may be that pandemic information campaigns on the internet mainly focuses on personal protection and transmission, while less attention is given to related academic issues. As college students pay more attention to the prevention and control information and measures during the outbreak, they don't know enough about COVID-19's knowledge level, which leads to the poor awareness rate of COVID-19's related academic questions. The results show that most students are aware of the common symptoms of COVID-19, such as fever, cough and dyspnea, but they do not know enough about diarrhea and other symptoms. At present, most COVID-19 cases are asymptomatic infections. In Segen's medical dictionary, asymptomatic infections are defined as those lacking obvious clinical symptoms. An epidemiological investigation shows that the spread of asymptomatic infections of COVID-19, which means that the human body has almost no symptoms after infection, but it can be found that it has been infected through antibody testing, may lead to serious clinical diseases (34), suggesting that publicity and education regarding atypical symptoms should be strengthened through effective channels. It was found that college students have poor knowledge of the correct way to cover their noses and mouths when coughing and sneezing, which suggests that there are still some shortcomings regarding personal protection against COVID-19. With the continuous development of intelligent technology, network information is more and more concerned and relied on by people, and it is more and more deeply involved in people's daily life. While the Internet is becoming more and more prosperous, it is inevitable that the information is difficult to distinguish between true and false. It is found that one of the reasons for this phenomenon is that, driven by economic interests, some people who lack the awareness of public morality and the bottom line of laws and regulations, for their own interests (mainly economic interests), do not hesitate to create and spread false information and sensational news by online channels. There are also some online media participants who, with some bad motives, irresponsibly create rumors and attack and hurt today's society or individuals out of thin air. Therefore, the government official website, as the main channel for authoritative information sources, needs to expand its publicity to compensate for the lack of information validation for online social tools. In addition, the popularization of accurate COVID-19 knowledge should be strengthened in relevant school courses, and popular science of infectious disease prevention education should be provided. The research showed that the awareness rate of male students' health knowledge was lower than that of female students. The reason may be that female students pay more attention to social health problems, are more aware about preventive measures and have a higher knowledge acquisition ability than male students, which is consistent with related research results (35–39). Studies by Rana et al. (40) showed that there are significant differences in risk perception and coping mechanisms between the sexes. Compared with those of males, the risk perceptions of females increased by 2.26%, and their coping mechanisms increased by 3.41%. Therefore, accurate health education should be provided among college students, and attention should be given to boys. The results showed that the awareness rate of medical college students was higher than that of non-medical college students, which may be because medical students have corresponding medical professional knowledge, consistent with the results of other studies (41, 42). In addition, the knowledge awareness rate of fifth-year college students was high, which may be related to the fact that fifth-year college students are all medical students. It is suggested that health education should be strengthened for non-medical students.

The survey results showed that college students had a positive attitude and were supportive of the prevention and control of COVID-19, contributing to the prevention and control of COVID-19. An early set of studies on the attitudes and knowledge of COVID-19 found that people's attitudes toward the government's measures to curb the pandemic were highly correlated with their level of knowledge of COVID-19 (43). It was found that a higher level of information and education was related to a more positive attitude toward COVID-19 preventive measures. In this study, females had higher attitude scores than males. Studies have found that young men are more likely to express feelings of being invincible to COVID-19, so effective public health suggestions must be formulated for them (44). Non-medical majors had higher attitude scores than medical majors, suggesting that medical students may experience the phenomenon of separation of knowledge, attitudes and practice.

The results showed that more than half of college students had high health behaviors (53.48%). Among them, urban college students had higher scores than rural college students. This may be because of the better implementation of protective measures in cities and towns, the lack of access to information in rural areas, and the limited access to information resources such as computers or social media that can help students understand the latest situation of the COVID-19 pandemic, suggesting that the education of relevant students should be strengthened (45). Medical students should continue to improve their cognition and attitude level, which play an important role in self-prevention measures and passing on knowledge to family, friends or relatives to help fight against epidemic diseases (46). The effective implementation of prevention and control measures is fundamental to curbing the spread of the pandemic. It was found that respondents had high execution ability in wearing masks, but the rate of good behaviors such as physical exercise was not high. At present, the COVID-19 pandemic is still spreading to different degrees. It is necessary to strengthen vaccination initiatives and continue to promote the existing guidelines for limiting the spread of the virus, including physical isolation, wearing masks, regular hand washing and indoor ventilation.

KAP theory emphasizes that knowledge is the foundation of behavioral change, attitude is the driving force of behavioral change, and knowledge improves attitudes, thus leading to a positive attitude toward change behaviors (47). Knowledge is a prerequisite for promoting healthy behaviors and developing a positive attitude to fight diseases (48). Providing training on COVID-19 prevention methods and publicizing correct information about the new coronavirus pneumonia will help to improve cognition and attitudes toward the prevention and control of COVID-19, which are essential to promote good COVID-19 prevention and control (49). Therefore, universities should plan to strengthen health education programs for COVID-19 prevention to educate students to take relevant preventive measures, such as maintaining social distance and avoiding gatherings in densely populated areas, and strengthen relevant publicity and education in a targeted manner; as a result, college students could develop a more comprehensive understanding of COVID-19, which is especially critical for enhancing their prevention and control behaviors.

### Interventions for college students' mental health should be considered

The research results showed that there were more boys than girls who had negative emotions. This may be related to the better awareness and attitudes of female college students regarding COVID-19. It was found that senior students had more obvious stress, anxiety and depression, which is similar to the results of the 2020 Chinese College Students' Health Survey Report. Logistics analysis of psychological status shows that ethnic Korean college students' scores of COVID-19's attitudes toward prevention and control are lower than those of Han college students, and ethnic Korean college students have more negative emotions than Han college students. It is suggested that lower levels of trust of ethnic Korean college students is the influencing factor of ethnic Korean college students' bad emotional status. In addition, in China, Chinese is the mother tongue for Han college students, while Chinese is the non-mother tongue for ethnic Korean college students, and ethnic Korean college students have poor understanding of knowledge. Therefore, compared with Han college students, ethnic Korean college students have lower levels of trust in prevention and control of COVID-19, and there are more negative emotional situations. The results suggest that schools should pay attention to the health education and psychological counseling of students in ethnic minority groups. In addition, the study found that a low KAP level was a risk factor for negative mood. The research subjects had a certain psychological burden in understanding the pandemic situation. Studies have found that college students may suffer more psychological troubles, such as stress and anger, due to isolation, school suspension, the prohibition of private gatherings and the reduction in learning efficiency due to online education, which leads to an increase in dangerous behaviors such as online gambling (50). As a public health emergency, the COVID-19 pandemic easily leads to individual psychological stress reactions, and stressful events are an important influencing factor of individual anxiety (51). In the era of high internet use, college students have strong insights into social problems, and when faced with stressful events, their emotional responses are more intense, and they are more likely to have problems such as depression, anxiety and stress (52). These results show that the mental health of college students cannot be ignored during the COVID-19 pandemic. Schools should instill a sense of social responsibility and community support in students, which may improve poor mental health outcomes. Colleges and universities should provide psychological counseling hotlines, and well-trained counselors and psychologists should provide free psychological intervention and counseling services through the Internet (53). Relevant school departments should provide scientific and effective publicity and psychological counseling, plan long-term psychological services to control and reduce the burden of psychological problems, and advocate for college students to increase physical exercise, develop good habits, and enhance their own resistance to formulate corresponding strategies and measures for prevention and control. In addition, schools should cooperate with government agencies and medical staff to conduct health promotion and information literacy projects to effectively improve the mental health level of college students (54). Considering that college students' psychological problems were previously widespread, COVID-19 will aggravate students' psychological problems, and the existing academic pressure and occupational pressure cannot be ignored. The results of this study can supplement information regarding the mental health status of college students in the COVID-19 period. In addition, the results of this study can also help to identify college students with increased risk of psychological problems. Universities can consider developing long-term psychological services, such as implementing KAP intervention measures regarding COVID-19, for these students to reduce their risk of psychological problems (55).

This research has some limitations. First, this study was limited to students in public universities, and online questionnaires were used. Not all college students in the whole school participated in this study. Therefore, the representativeness of the research results to all students is controversial. In addition, the data provided in this study were self-reported, so there may be recall bias. Due to the cross-sectional study design, the viewpoint may change, and this study is also unable to establish causality.

## Conclusion

College students in minority areas have good health attitudes, but the awareness rate of COVID-19 prevention and control is low, the proportion of healthy behaviors is low, and negative emotions easily occur. It is suggested that schools and other departments adopt relevant KAP intervention strategies, further strengthen targeted publicity and education measures, and provide relevant psychological counseling to reduce the harm of the COVID-19 pandemic to health.

## Data availability statement

The datasets presented in this article are not readily available to avoid misuse of data and information, the datasets used or analyzed during the current study are available from the corresponding author Y-HL on reasonable request. Requests to access the datasets should be directed to Y-HL, liyihua@ybu.edu.cn.

## Ethics statement

The studies involving human participants were reviewed and approved by Institutional Review Board of Yanbian University (Ethical Code: 2021576, October 26th, 2021). Written informed consent for participation was not required for this study in accordance with the national legislation and the institutional requirements.

## Author contributions

Conceptualization and formal analysis: TW, Y-HL, and Y-QL. Data curation, methodology, and writing—original draft: TW and Y-HL. Investigation: Y-HL, Z-HH, Y-SC, and Y-QL. Supervision and validation: Y-HL, Y-SC, and Z-HH. Writing—review and editing: Y-HL and Y-QL. All authors contributed to the article and approved the submitted version.
